# Supplementary material for: Overcoming Resistance to Immune Checkpoint Inhibitor Therapy Using Calreticulin-Inducing Nanoparticle
Source: Pharmaceutics. 2023 Jun 9;15(6):1693. doi: 10.3390/pharmaceutics15061693 (PMC10302072; doi:10.3390/pharmaceutics15061693)
Supplement: Supplementary file 1 [file pharmaceutics-15-01693-s001.zip › pharmaceutics-2318808-supplementary.pdf]

Supplementary Figure:

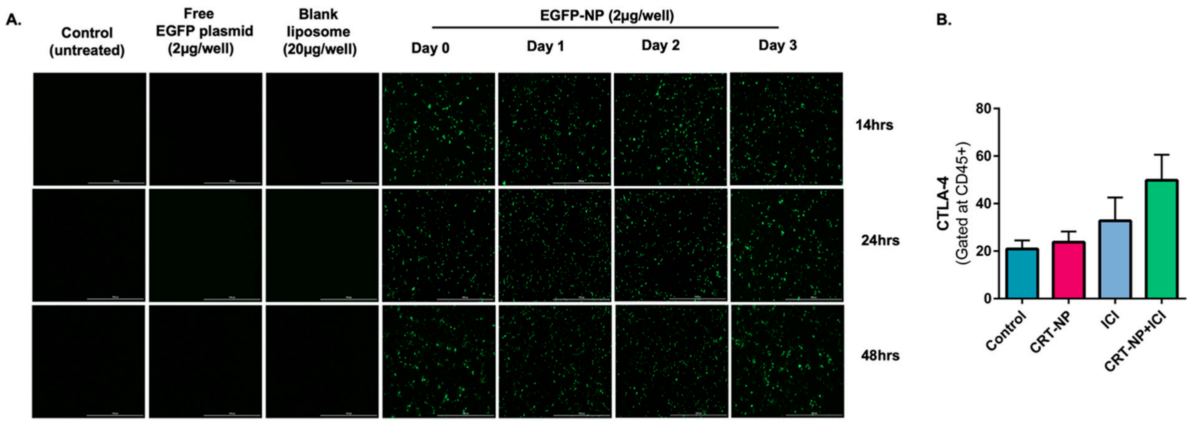

Figure S1. (A) CRT-NP induced stable transfection efficiency for several days. CT26 cells were transfected with EGFP-NPs stored at 4°C on the synthesis day (day 0), and subsequently, the same batch was used to transfect cells on the second day (day 1), third day (day 2), and fourth day (day 3) post particle synthesis. Fluorescence imaging at 14, 24, and 48 h post transfection indicated similar GFP expression rates, suggesting the efficient plasmid encapsulation and stability of the NPs. (B) Expression of CTLA4 on CD45+ immune cells showed enhanced expression with the combination treatment; however, there were no significant differences observed between the various treatment groups.
